# Ocular Syphilis in an HIV-Positive Transgender Female: A Case Report

**DOI:** 10.7759/cureus.66775

**Published:** 2024-08-13

**Authors:** Kyle J Roe, Colton P Boney, Unika Mirza, Robert W Parker

**Affiliations:** 1 Medicine, Alabama College of Osteopathic Medicine, Dothan, USA; 2 Medicine, Alabama College of Osteopathic Medicine, Huntsville, USA; 3 Pharmacology, Alabama College of Osteopathic Medicine, Dothan, USA

**Keywords:** gender-affirming care, lgbtq, sexually transmitted infection (sti), syphilis, treponema pallidum, hiv care, transgender care, ocular syphilis

## Abstract

This is a case of a 56-year-old transgender female with a history of HIV who presented to the emergency department with visual disturbances and bilateral papilledema. Initially, intracranial abnormalities were ruled out through imaging studies. However, a lumbar puncture later confirmed the presence of syphilis in the cerebral spinal fluid (CSF), and the patient was diagnosed with bilateral syphilitic uveitis by a retina specialist. Treatment with intravenous and intramuscular penicillin led to significant improvement in her visual symptoms and resolution of optic nerve edema. This case underscores the importance of early screening for syphilis and other sexually transmitted infections (STIs) in transgender patients living with HIV presenting with visual symptoms. The delayed syphilis screening and treatment in this patient highlight the impact of healthcare barriers on transgender individuals. Prompt diagnosis and treatment are critical to prevent serious complications, such as permanent vision loss. Healthcare providers must maintain a high index of suspicion for syphilis in HIV-positive patients with visual symptoms, irrespective of their cluster of differentiation 4 (CD4) count or viral load. Addressing barriers to healthcare for transgender individuals is essential to ensure timely diagnosis and treatment to improve patient outcomes.

## Introduction

Transgender patients are more likely to encounter barriers in seeking healthcare than their cisgender counterparts. These barriers can often lead to patients delaying necessary care, which can have a profound impact on their lives. In the Southeastern United States, in particular, these barriers are multifactorial, including patient fear or mistrust of providers and mistreatment due to their gender identity [1]. Transgender persons, especially transgender women, are at a much higher risk than their cisgender counterparts of contracting HIV and other sexually transmitted infections (STIs), including syphilis [2]. When combined with the barriers to seeking care, the higher risk of transgender patients contracting these infections poses a risk to their overall health. Both HIV and STIs are very treatable and manageable conditions when diagnosed early. However, the longer that patients go without being diagnosed and receiving treatment, the more likely they can incur severe complications that may require more extensive treatment. This extensive, delayed treatment can also cause an increase in the overall cost of care for these patients as more tests, procedures, or lengthy hospital stays are necessary to ensure that they receive the appropriate treatment [3,4].

## Case presentation

The patient was a 56-year-old transgender female who went to her ophthalmologist with a chief complaint of "seeing smoke." On examination of the patient, the ophthalmologist found bilateral papilledema, prompting immediate referral to the emergency department (ED) for inpatient assessment. During evaluation in the ED, the patient reported having a mild headache around the occipital lobe, which she described as a head pressure sensation that had been present for several days prior. She also reported intermittent photosensitivity and blurred vision. Her past medical history was significant for HIV infection diagnosed one year prior. A review of the systems was negative for skin changes, fever, or chills. The patient also denied traveling outside Alabama or knowledge of any recent sick contacts. An infectious disease specialist was managing her HIV infection, and the patient reported being adherent to her medications. Her current HIV antiretroviral therapy (ART) was one dolutegravir/lamivudine (50/300 mg) tablet taken once daily. Her last cluster of differentiation 4 (CD4) count was 332 cells/mm^3^, and she had an undetectable viral load. She reported having multiple sexual partners since her HIV diagnosis. Her blood pressure was 125/71 mmHg, her heart rate was 61 beats per minute, and she was afebrile. An eye exam noted clear conjunctiva and sclera. No deficit of cranial nerves II-XII was found. 

Her complete blood count and comprehensive metabolic panel showed elevated creatinine levels and leukopenia. The results of her relevant labs are presented in Table 1. A computed tomography (CT) of the head without contrast, CT angiogram of the head, magnetic resonance imaging (MRI) of the brain with and without contrast, and MRI of the face, neck, and orbit with and without contrast were ordered to rule out intracranial abnormalities. These tests were negative for any acute abnormalities, including optic neuritis. Magnetic resonance cerebral venography was also ordered. This test showed no dural venous sinus thrombosis in the right dominant venous drainage system. Based on the patient's previous medical and social history, a syphilis rapid plasma reagin (RPR) test was ordered and came back positive with a titer value of 1:128 (<1:1). A lumbar puncture was ordered to test cerebral spinal fluid (CSF) for syphilis in order to rule out neurosyphilis. The CSF results came back positive for syphilis antibody. Additional CSF results are reported in Table 1. An infectious disease specialist was consulted, who made a referral to a retina specialist for the patient to see post-discharge. The patient had a central line placed and received one 4 million unit dose of penicillin infusion over six hours during her hospital stay. She was discharged after a 72-hour stay with no antibiotics.

**Table 1:** Lab work WBC: White blood cell; RBC: Red blood cell; VDRL: Venereal disease research laboratory; CSF: Cerebrospinal fluid; RPR: Rapid plasma reagin

Test	Patient’s results	Reference range
WBC count	3.49	4.10-12.20 x 10^3^/uL
RBC count	3.4	4.7-6.1 x 10^6^/uL
Hemoglobin	10.9	13.5-17.5 g/dL
Hematocrit	33	41-50%
Platelet count	130	137-352 x 10^3^/uL
Creatinine level	1.3	0.7-1.2 mg/dL
VDRL, CSF	Positive	Negative
WBC count, CSF	8	<20 cells/mL
Protein count, CSF	52	15-45 mg/dL
RPR titer	1:128	<1:1

The day after discharge from the hospital, the patient was seen by the retina specialist. Visual acuity assessment showed 20/60 (20/20) on the right and 20/50 (20/20) on the left. The patient was noted to be wearing a contact lens in her left eye during the visual acuity assessment. The retina specialist diagnosed the patient with bilateral syphilitic uveitis. An ocular exam showed evidence of active inflammation with 2+ cells in the vitreous humor of the right eye, trace cells in the vitreous humor of the left eye, and optic nerve edema. The retina specialist recommended that the patient schedule a follow-up appointment with the infectious disease specialist during her hospital stay to continue treatment. The patient was scheduled for a follow-up with the retina specialist in one week.

Two days after hospital discharge, the patient had an appointment with the infectious disease specialist. The specialist wrote an order for antibiotic therapy based on the syphilitic uveitis diagnosis and put the patient in contact with an outpatient infusion service to begin her intravenous (IV) penicillin course of 4 million units over six hours for 14 days. Once the IV infusion regimen was complete, the patient was to start 2.4 million units of intramuscular (IM) benzathine penicillin once weekly for three weeks. At a one-week follow-up, the infectious disease specialist reported that the patient tolerated the medication well. The patient noted that her vision was improving and no longer reported photophobia. The infectious disease specialist noted that the current treatment should continue as planned and that the patient should be seen again for a follow-up in one week. At one week follow-up with ophthalmology, the patient's visual acuity improved to 20/40 (20/20) on the right and 20/30 (20/20) on the left. On ocular examination, no active inflammation was present in either eye, and the optic nerve edema had improved. At an eight-week follow-up appointment with ophthalmology, the patient's visual acuity continued to improve to 20/30 (20/20) on the right and 20/40 (20/20) on the left. On ocular examination, the patient's optic nerve edema had resolved. At a six-month follow-up with infectious disease, the patient had an RPR titer value of 1:16 and did not report any recurrence of symptoms.

## Discussion

Syphilis is a sexually transmitted infection caused by the bacteria *Treponema pallidum* and occurs in four stages: primary, secondary, tertiary, and latent. Neurosyphilis, ocular, and otic syphilis can occur at any stage of infection, with different clinical presentations for each [5]. In patients with HIV, primary or secondary syphilis may also cause a transient decrease in CD4 cell count and an increase in HIV viral load that improves with syphilis treatment [6]. Coinfection with HIV has also shown an increase in the progression of syphilis infection and may alter its clinical presentation [7]. This disease progression makes it important for healthcare providers to be aware of it in the HIV patient population. Patients living with HIV are also at a higher risk of contracting STIs, including syphilis. Current guidelines are to test for STIs upon initial HIV-related care and then repeat annually; however, if patients present with other risk factors, this testing frequency should be increased [8,9]. These risk factors include having sex with multiple partners, having sex in exchange for money or other goods and services, and having sex with methamphetamine use [10]. In these patients, testing is recommended every three months to diagnose syphilis and other STIs at earlier stages to prevent serious complications.

While syphilis can involve almost any ocular structure, the most common are posterior uveitis and panuveitis. It is recommended that all patients with ocular symptoms and reactive syphilis serology receive an ophthalmologic examination that includes a slit lamp examination and cranial nerve evaluation [11]. If cranial nerve dysfunction is noted, CSF evaluation is needed. In patients with only ocular symptoms, reactive syphilitic serology, ocular examination abnormalities, and a normal neurological exam, CSF examination is not needed. Treatment should also begin promptly once the diagnosis of ocular syphilis is suspected. Making a diagnosis of ocular syphilis requires a high index of suspicion and serologic confirmation of syphilis while also ruling out other causes of vision loss [12]. However, an ocular syphilis diagnosis should be ruled out in any HIV patient presenting with visual symptoms regardless of CD4 count or HIV viral load [7]. Radiological imaging was used in this patient to rule out intracranial and vascular causes of vision loss. A positive syphilis result should also warrant screening for other STIs to guide the appropriate treatment plan. Given the risk of permanent vision loss, cost of further testing, and coinfection rates for those living with HIV, clinicians should have a low threshold to order a syphilis test for patients presenting with these symptoms.

Treatment of ocular syphilis is the same as neurosyphilis. The recommended treatment is 18-24 million units per day of aqueous crystalline penicillin G, which should be administered IV in 3-4 million units over four hours or as continuous IV infusion for 10-14 days [13]. The regimen for asymptomatic neurosyphilis, regardless of HIV status, is 2.4 million units of benzathine penicillin G given intramuscularly once weekly for three weeks. In patients with a neurosyphilis diagnosis, a slower serologic response to treatment is often expected.

For the patient presented in our case report, the suspicion of ocular syphilis should have been high, given the patient’s HIV status and social history regarding multiple sexual partners. The patient also met criteria that warranted a full STI panel upon visiting the ED and during her hospital stay. Transgender patients often express fear when disclosing their gender identity to healthcare professionals due to anticipation of discrimination and suboptimal or inappropriate care [4]. This fear and anxiety start from the moment the patient visits registration, encountering obstacles such as insensitive staff or electronic health records and patient intake forms. While the steps taken to rule out other causes of vision loss were appropriate and warranted, the patient did not receive a syphilis test until 48 hours after hospital admission. The patient in our case report also received a lumbar puncture to evaluate for neurosyphilis. However, current guidelines indicate this is unnecessary unless neurologic deficits exist [6,14]. For this patient, cranial nerve function was reported to be normal. In HIV-positive patients, a CSF evaluation is recommended with an RPR titer level of 1:32 or higher, a CD4 count of 350 cells/mm^3^ or lower, and an absence of ART [15]. When the lumbar puncture was done, the RPR titer results had not yet returned from the lab. While her latest CD4 count of 332 cells/mm^3^ met the criteria (<350 cells/mm^3^) to perform a CSF examination, her compliance with ART may have excluded her from a lumbar puncture based on the guidelines for HIV-positive patients. The CSF results were also not needed to start treatment in this patient for syphilis infection.

When compared to another case report of ocular syphilis involving a cisgender male, a CSF examination was not performed. He also received tests in addition to the syphilis screening, including a hepatitis panel, gonorrhea, and chlamydia, to rule out other potential infections [15]. This patient was also admitted to the hospital, similar to the transgender female patient presented in our case report, and radiological imagery was performed as well. However, this male patient began his IM penicillin treatment during his hospitalization. While the patient in our case report was given a single dose of penicillin before discharge, she did not begin her IV infusion penicillin treatment until 48 hours post-discharge. While these patients were not treated in the same hospital or by the same clinical staff, it may highlight the experience that has been reported by transgender patients in previous research studies [16]. The patient presented in our case report required several follow-up appointments to begin receiving treatment that could have been coordinated with an outpatient infusion center prior to her discharge. While the patient in our case report complied and attended each follow-up appointment, this is not always true. By not administering treatment promptly, a patient with a similar presentation may forgo these crucial follow-up visits and increase their risk of severe complications from ocular syphilis, such as permanent vision loss. 

## Conclusions

This case highlights the critical need for prompt and comprehensive screening for syphilis and other sexually transmitted infections in HIV-positive transgender patients presenting with visual abnormalities. Despite initial delays in diagnosis, appropriate treatment with intravenous and intramuscular penicillin significantly improved the patient's condition. This case underscores the importance of healthcare providers maintaining a high index of suspicion for ocular syphilis in similar patients and addressing barriers to healthcare access that transgender individuals often face. Early diagnosis and treatment are crucial to prevent severe complications and improve patient outcomes as cases of syphilis continue to rise in the United States. 
